# Antibody Signature of Spontaneous Clearance of *Chlamydia trachomatis* Ocular Infection and Partial Resistance against Re-challenge in a Nonhuman Primate Trachoma Model

**DOI:** 10.1371/journal.pntd.0002248

**Published:** 2013-05-30

**Authors:** Laszlo Kari, Lauren E. Bakios, Morgan M. Goheen, Leah N. Bess, Heather S. Watkins, Timothy R. Southern, Lihua Song, William M. Whitmire, Norma Olivares-Zavaleta, Harlan D. Caldwell

**Affiliations:** Laboratory of Intracellular Parasites, Rocky Mountain Laboratories, National Institute of Allergy and Infectious Diseases, National Institutes of Health, Hamilton, Montana, United States of America; University of California San Francisco, United States of America

## Abstract

**Background:**

*Chlamydia trachomatis* is the etiological agent of trachoma the world's leading cause of infectious blindness. Here, we investigate whether protracted clearance of a primary infection in nonhuman primates is attributable to antigenic variation or related to the maturation of the anti-chlamydial humoral immune response specific to chlamydial antigens.

**Methodology/Principal Findings:**

Genomic sequencing of organisms isolated throughout the protracted primary infection revealed that antigenic variation was not related to the inability of monkeys to efficiently resolve their infection. To explore the maturation of the humoral immune response as a possible reason for delayed clearance, sera were analyzed by radioimmunoprecipitation using intrinsically radio-labeled antigens prepared under non-denaturing conditions. Antibody recognition was restricted to the antigenically variable major outer membrane protein (MOMP) and a few antigenically conserved antigens. Recognition of MOMP occurred early post-infection and correlated with reduction in infectious ocular burdens but not with infection eradication. In contrast, antibody recognition of conserved antigens, identified as PmpD, Hsp60, CPAF and Pgp3, appeared late and correlated with infection eradication. Partial immunity to re-challenge was associated with a discernible antibody recall response against all antigens. Antibody recognition of PmpD and CPAF was destroyed by heat treatment while MOMP and Pgp3 were partially affected, indicating that antibody specific to conformational epitopes on these proteins may be important to protective immunity.

**Conclusions/Significance:**

Our findings suggest that delayed clearance of chlamydial infection in NHP is not the result of antigenic variation but rather a consequence of the gradual maturation of the *C. trachomatis* antigen-specific humoral immune response. However, we cannot conclude that antibodies specific for these proteins play the primary role in host protective immunity as they could be surrogate markers of T cell immunity. Collectively, our results argue that an efficacious subunit trachoma vaccine might require a combination of these antigens delivered in their native conformation.

## Author Summary

*Chlamydia trachomatis* is the etiological agent of trachoma the world's leading cause of infectious blindness. In this study, we investigated whether delayed clearance of a primary infection in nonhuman primates was attributable to antigenic variation or related to gradual changes in the humoral immune response specific to chlamydial antigens. We found that antigenic variation was not related to the inability of monkeys to efficiently resolve their infection. However, exploring changes in the immune response as a possible reason for delayed clearance revealed that antibody recognition was restricted to the antigenically variable major surface protein and a few conserved polypeptides. Antibody recognition of the major antigenically variable surface protein correlated with the initial reduction in infectious burdens while recognition of conserved chlamydial antigens occurred late and correlated with infection eradication. These findings suggest that delayed clearance of chlamydial infection is not the result of antigenic variation but a consequence of a gradually evolving humoral immune response specific to different chlamydial antigens. Antibody recognition was at least partially directed against conformational epitopes, indicating that an efficacious subunit trachoma vaccine might require a combination of antigens delivered in their native conformation.

## Introduction

The obligate intracellular bacterial parasite *Chlamydia trachomatis* is the causative agent of blinding trachoma and sexually transmitted diseases. *C. trachomatis* utilizes a unique biphasic developmental cycle alternating between infectious elementary bodies (EB) and metabolically active reticulate bodies (RB). Multiple serovars exist within *C. trachomatis*. The *ompA* gene, coding for the immunodominant major outer membrane protein (MOMP), differentiates these serovars [1]. Serovars A, B, Ba, and C are the etiological agents of trachoma [2], the global impact of which is significant. Designated by the WHO as one of the major neglected tropical diseases [3] it is the world's leading cause of preventable blindness, primarily afflicting populations in developing nations [4]. Where endemic, trachoma infection is initiated at a very early age presenting as acute follicular conjunctivitis. However, prolonged repeated infection due to deficient protective immunity can trigger chronic pro-inflammatory immune responses leading to conjunctival scarring, trichiasis, and corneal opacity. Though chronicity of infection is frequently believed to relate to constant exposure and reinfection, the pathogenesis of trachoma is not fully understood. It is believed that an imbalance of host protective and pathological immune response is responsible for the pathophysiology of the disease. Poor natural immunity leads to multiple bouts of re-infection that serve as the antigenic stimulus for a sustained damaging inflammatory pathologic immune response [4]. Uncertainty remains however regarding the precise contribution of long-duration chlamydial infections and reactivations in trachoma pathology.

The nonhuman primate ocular model is the most relevant animal model for studying trachoma. Not only is this ocular model appropriate in its ability to mimic the acute aspects of human trachoma infection but isolation of laboratory animals ensures infection exposure and disease are not related to reinfection. Previously, we used this model to examine infection with a recently isolated virulent Tanzanian clinical strain of *C. trachomatis* serovar A, A2497 [5]. We reported that following ocular infection of cynomolgus monkeys, an initial peak shedding period was followed by clearance and bouts of smaller reactivation peaks of infection that lasted for months. Clinical response scores of hyperemia and follicle formation remained high throughout the infection period and continued for weeks after complete absence of bacterial shedding. This experimental picture in NHP closely mimics the acute phase of the naturally occurring infection in hyperendemic trachoma regions.

In this study, we investigate whether antigenic drift or maturation of the host *C. trachomatis* specific humoral immune response might explain the basis of the protracted period of time required to eradicate the primary infection and clinical disease. We found no evidence linking antigenic variation to delayed clearance of primary infection in these animals but observed convincing findings implicating gradual changes in the humoral immune response specific to a few chlamydial antigens as a possible mechanism.

## Materials and Methods

### Ethics statement

Healthy adult naïve cynomolgus macaques (*Macaca fascicularis*) maintained at the Rocky Mountain Laboratories were cared for under standard practices implemented by the Rocky Mountain Veterinary Branch/NIAID/NIH. Monkeys were housed separately when being used for experimental studies. All handling procedures were reviewed and affirmed by the Animal Care and Use Committee at Rocky Mountain Laboratories and work was conducted in full compliance with the Guide for Care and Use of Laboratory Animals. The facilities are fully accredited by the American Association for Accreditation of Laboratory Animal Care. Conjunctival chlamydial infections can cause transient local inflammation in the eye and are not associated with pain or significant discomfort; discomfort is usually limited to minor photophobia. During the experimentations we were prepared to treat any animals determined to be under stress or in significant discomfort with appropriate analgesics and antibiotics. We observed no stress or significant discomfort during the entire experiment. At the end of the nonhuman primate re-challenge experiment animals were treated with antibiotics and returned to their colony unharmed.

### Chlamydial strains and propagation

*C. trachomatis* strains A2497 (serovar A) and Ba/Ap-2/OT (serovar Ba) were cultured in either HeLa 229 or McCoy cells (ATCC) and elementary bodies (EBs) were purified by density gradient centrifugation [6].

### Infection of NHPs

All NHP ocular chlamydial infections and re-infections were done using 2×10^4^ IFU per eye directly applied on the conjunctival surface of the upper and lower lids of both eyes with strain A2497. Chlamydial culture and ocular clinical disease scoring was done as previously described [7]. Briefly, swabs were taken from the upper and lower conjunctiva of each eye, and chlamydiae were cultured on HeLa cells to monitor infection. Each eye was scored for disease based on both the intensity of conjunctival hyperemia and follicle formation. Hyperemia and follicular scoring were combined to produce an aggregate clinical disease score shown graphically on a scale of 0–12 with 12 being the maximum score. Blood was collected at the same time as culturing and clinical scoring.

### Experimental design

Three male cynomolgus monkeys were infected ocularly with the A2497 trachoma strain and the infections were allowed to run their natural self-limiting course (4–6 months). Approximately three months following spontaneous clearance of infection and disease (269 days post primary infections) monkeys were similarly re-challenged and again the infections were allowed to run their natural course. At weekly intervals during the entire experiment, ocular disease was evaluated and scored and samples were collected. The primary infection of these animals has been described before in the *Journal of Infectious Diseases* [7].

### *ompA* sequencing of plaque cloned isolates

At various times post-infection chlamydiae cultured from the conjunctivae of all three monkeys were plaque cloned using McCoy cells [8]. For each isolate 20–24 individual plaques were selected and consecutively passed twice in McCoy cells. DNA was isolated from chlamydial cells 48 hours post infection (hpi) by lysis in 0.2 ml of 0.5 M NaOH and then neutralized by the addition of 0.2 ml of 1M Tris-HCl pH 8.0. *ompA* PCR and sequencing was performed as previously described [7]. Sequencing data, in FASTA format, was compiled for each original clone with the CAP3 Sequence Assembly Program (http://pbil.univ-lyon1.fr/cap3.php). Using the NCBI website (http://www.ncbi.nlm.nih.gov/blast/bl2seq/wblast2.cgi), assembled sequences were compared against the previously reported A2497 *ompA* sequence [7].

### Genome sequencing of plaque cloned isolates

Plaque clones from monkey RML126 were passed in HeLa 229 cells to obtain sufficient organisms to infect six well culture plates. Six 6-well culture plates of HeLa cells were infected with each clone at an MOI of 1. At 48 hpi, chlamydiae were harvested and partially purified on 30% MD-76R (Merry X-Ray Corporation) density gradients [6]. Genomic DNA was isolated from 10^8^ organisms as previously described [9]. Ten µg of DNA for each clone was used for genome sequencing by NimbleGen Systems as previously described [7].

### Intrinsic radiolabeling of chlamydiae

McCoy cells grown in 6 well tissue culture plates were infected with *C. trachomatis* serovar A or serovar Ba at a MOI of 2.5 and fed with RPMI (supplemented with 1% L-glutamine, 0.33% glucose, 10% dialyzed FBS, and gentamicin) containing 4 µg/ml of emetine. Twelve hpi cells were washed twice with 1 ml of RPMI minus L-methionine and L-cysteine and then incubated with 300 µCi of ^35^S L-methionine and L-cysteine in 3 ml of RPMI. At 50 hpi the monolayers were washed 3 times with 5 ml PBS and then lysed in 1 ml of cold RIPA lysis buffer (25 mM Tris-HCl pH 7.6, 150 mM NaCl, 1% NP-40, 1% sodium deoxycholate, and 0.1% SDS) containing protease inhibitor (Roche Protease Inhibitor Cocktail Tablet). The cell lysate was gently passed through a 25 gauge needle 10 times to shear DNA and centrifuged at 19,000× g at 4°C for 30 min. Intrinsically radiolabelled antigen supernatants were collected, aliquoted, and stored at −20°C, with aliquots used in immunoassays.

### Radioimmunoprecipitation (RIP) assay

Fifty µl Protein A and 50 µl Protein G magnetic beads (Invitrogen) were used for each RIP reaction according to the manufacturer's protocol. Briefly, beads were washed several times with Ab Binding and Wash Buffer. Intrinsically radiolabeled chlamydial antigen lysates were pre-cleared by incubation with 100 µl of beads for 1 hr at RT with continuous rotation. Beads were then re-suspended in Ab Binding and Wash Buffer. NHP sera were added to the suspension to achieve a final dilution of 1∶80. Tubes were rotated for 1 hr at RT and the supernatants removed. Beads were washed and then incubated with 100 µl of pre-cleared antigen lysate for 2 hours by rotating at RT. Supernatants were removed and the beads were washed four times and then transferred to a clean tube. Beads were suspended in 30 µl of 2× Laemmlie sample buffer (BioRad) and boiled for 10 min at 100°C. Supernatants were removed from the beads, loaded onto 4–15% precast SDS-PAGE gels (BioRad), and electrophoresed at 70 V for 30 min and then 150 V for 70 min. Gels were rinsed in 5 ml of dH_2_O for 10 min and then placed on filter paper, vacuum dried and exposed to high performance chemiluminescence film (Amersham Hyperfilm ECL).

### Western blotting of immunoprecipitated non-radiolabeled chlamydial infected cells

McCoy cells were infected with chlamydiae and chlamydial antigen lysates were prepared as described above for intrinsically radiolabeling experiments, except no ^35^S L-methionine and L-cysteine was added to the culture media. The immunoprecipitation (IP) procedure used was also identical with the exception that the final elution of antigen bound to beads employed the Invitrogen non-denaturing elution protocol using 20 µl elution buffer for 5 min at RT. The elution supernatant was transferred to a new tube and 20 µl of 2× sample buffer was added to each tube. Samples were boiled for 10 min at 100°C and electrophoresed as described above. Proteins were transferred to PVDF (BioRad) membranes for 4 hr at 350 mA. Following transfer, the membranes were blocked overnight, washed twice with diH20, and incubated with mouse monoclonal or rabbit monospecific primary antibody specific to chlamydial proteins at RT for 3 hours. The membrane was washed, incubated with anti-mouse or anti-rabbit secondary antibody at RT for 1 hr, washed repeatedly, and then incubated with 1 ml of chemiluminescent developing solution for 5 mins at RT (Invitrogen). Gels were exposed to high performance chemiluminescence film (Amersham Hyperfilm ECL).

## Results

### Ocular infection and re-challenge of nonhuman primates

In our previous study three cynomolgus monkeys were infected ocularly with *C. trachomatis* strain A2497, a recent trachoma clinical isolate with enhanced virulence characteristics for the nonhuman primate (NHP) eye [7]. Primary ocular infection continued for up to 14 weeks post infection (wpi) and then resolved spontaneously (RML134: 13 wpi; RML124: 8 wpi; RML126: 14 wpi). Monkeys were partially immune to re-challenge 3 months following clearance of the primary infection and disease. Re-challenged monkeys shed significantly less chlamydiae for a much shorter duration (RML134: 3 wpi; RML124: 5 wpi; RML126: 7 wpi) accompanied by reduced ocular disease that was also of shorter duration than that observed for the primary infections (11 wpi versus 30 wpi, respectively). Interestingly, the initial 4–5 weeks of the primary infection was characterized by higher levels of bacterial shedding, whereas later time points (6–14 wpi) were characterized by fluctuating periods of culture positive and negative results with much less organism being shed from the conjunctivae. Intense ocular disease was present in two of the three animals (RML 134 and RML 126) during this period of low infectious burden and an additional 15 weeks was required for disease resolution despite being culture negative for chlamydiae.

### Delayed clearance of primary ocular infection is not attributable to antigenic drift

To examine the possible role of antigenic drift in delayed clearance, chlamydia positive ocular swabs from time points following culture negative periods were cultured and chlamydiae were plaque cloned (RML134: week 9 and 12; RML124: week 8; RML126: week 8, 11 and 14). We initially looked for antigenic variation in *ompA*, the gene encoding the major outer membrane protein (MOMP), an immunodominant neutralizing target and the serotyping antigen of chlamydiae [1], [6]. We examined over 200 re-isolated clones but no *ompA* sequence differences were found (data not shown). To look for antigenic variation beyond *ompA*, 4 clones from the final reactivation period (week 14) and 2 clones from the initial peak shedding period (week 1) in one monkey (RML 126) underwent comparative genomic sequencing. As with *ompA*, no genetic variation was observed between the genomes of the plaque clones isolated from early and late time points (data not shown).

### Antibody signature of *C. trachomatis* infected nonhuman primates

**Figure 1:**
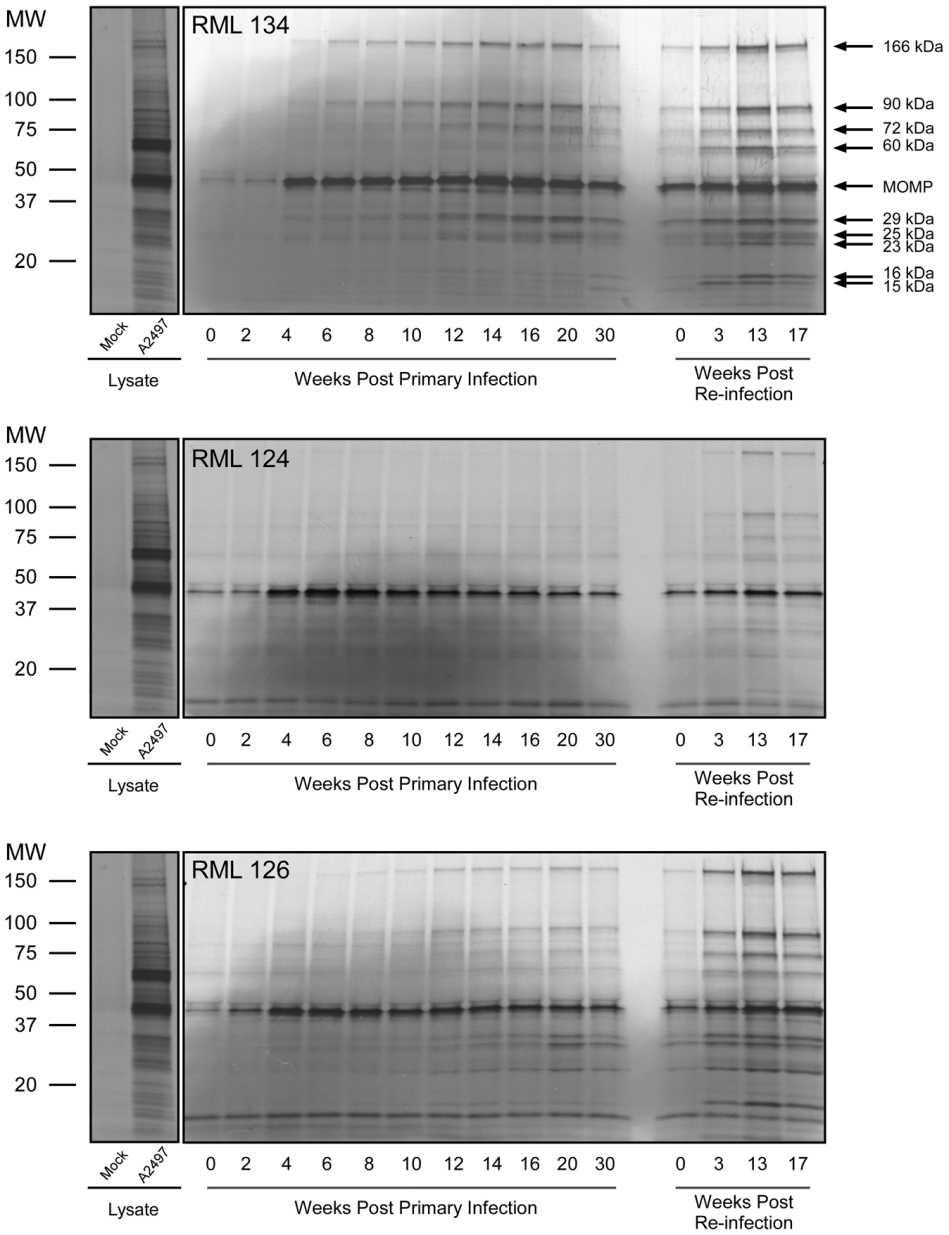
Antibody signature of *C. trachomatis* infected macaques following primary infection and re-challenge. Three monkeys were infected ocularly with *C. trachomatis* strain A2497 [7]. Monkeys cleared the infection 9–14 wpi (RML134: 13 wpi; RML124: 8 wpi; RML126: 14 wpi). Clinical disease remained severe to moderately severe during the periods of culture positivity and continued until about 30 wpi. Three months after resolution of disease monkeys were re-challenged and all monkeys exhibited partial protection characterized by a more rapid clearance of organism (3–7 wpi) with an accompanying reduction in the intensity and duration of ocular inflammation (11 wpi). Sera collected from monkeys during primary infection and following re-challenge were analyzed by RIP using intrinsically labeled chlamydial proteins prepared from non-denaturing detergent lysed infected McCoy cells. Sera from all three macaques produced a similar yet simple antigen recognition profile that differed in complexity over the period of primary infection and re-challenge. A total of 10 proteins were immunoprecipitated with molecular masses of 166, 90, 72, 60, 40, 29, 25, 23, 16 and 15 kDa. There was a noticeable temporal and recall recognition pattern of the antigens in all three monkeys. The MOMP (40 kDa) recognition was strong very early post infection (2–4 weeks) and consistently sustained throughout the entire period of infection and disease. In contrast immunoprecipitation of the other chlamydial proteins occurred later following primary infection, with the strongest immunoprecipitation reactions occurring at the time of infection eradication and disease resolution. There was a recognizable increase in the immunoprecipitation of all of these proteins by all three animals following re-challenge.

To investigate the possible role of an evolving antibody mediated immune response we collected sera from the three monkeys during primary infection and following re-challenge. These sera were analyzed by radioimmunoprecipitation (RIP) with intrinsically radiolabelled labeled chlamydial proteins prepared from non-denaturing detergent lysed infected cells. Despite the complex composition of the lysates, we observed a surprisingly simple antibody recognition profile by RIP that was consistent among the three nonhuman primates (Figure 1). All three monkeys recognized the MOMP, a 40 kDa protein, and nine other polypeptides. The molecular weights of these proteins were 166, 90, 72, 60, and approximately 29, 25, 23, 16 and 15 kDa. There was a noticeable temporal and recall recognition pattern of the antigens by all three monkeys. The dominant MOMP was recognized early in infection (2–4 weeks) with sustained response through spontaneous clearance of the primary infection. Antibody recognition of other proteins occurred later during primary infection and peaked at the time of spontaneous resolution. We also observed a marked increase in recognition of all proteins following ocular re-challenge.

### Antibody specificity of NHP sera

**Figure 2:**
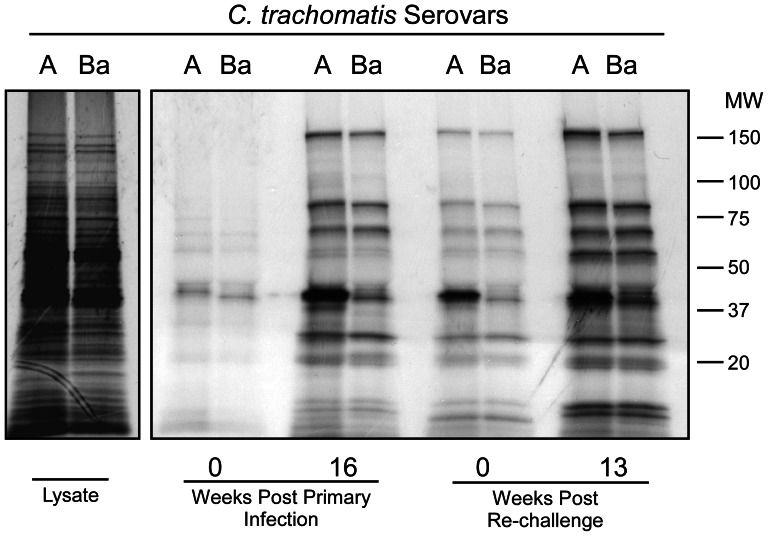
The majority of chlamydial antigens recognized during primary infection and re-challenge are shared between trachoma serovars. RIP was performed using lysates made from *C. trachomatis* serovars A2497 and Ba. Serum from monkey RML134 was used in the experiment and four representative time points throughout the course of infection and re-challenge were analyzed. Antibody to MOMP was serovar-specific, reacting most intensely with the infecting A2497strain. Conversely, antibody reactivity against the other immunogenic proteins was cross-reactive between trachoma serovars demonstrating that these antigens are conserved.

In order to investigate whether NHP antibodies recognize conserved or variable chlamydial antigens, RIP was performed using lysates made from *C. trachomatis* serovars A2497 and serovar B (Ba/Ap-2), strains differentiated by *ompA* antigenic variation that are representative of the C and B complex serogroups, respectively. For this experiment we used sera from a single monkey (RML 134) obtained at four representative timepoints: at the days of infection and re-challenge, and at the peak of anti-MOMP reactivity post infection and re-challenge (Figure 1). This monkey recognized the A2497 MOMP strongly but reacted poorly to the serovar B MOMP (Figure 2). In contrast, antibodies from this monkey reacted equally well to the 166, 90, 72, 60, 29 kDa and lower MW proteins demonstrating that they are conserved antigens shared between these two serologically distinct strains. Serum antibodies against these proteins diminished significantly four months following resolution of the primary infection but were clearly recalled following re-challenge and correlated with the partial immune status of the animal.

### Identification of conserved antigens by western blotting

**Figure 3:**
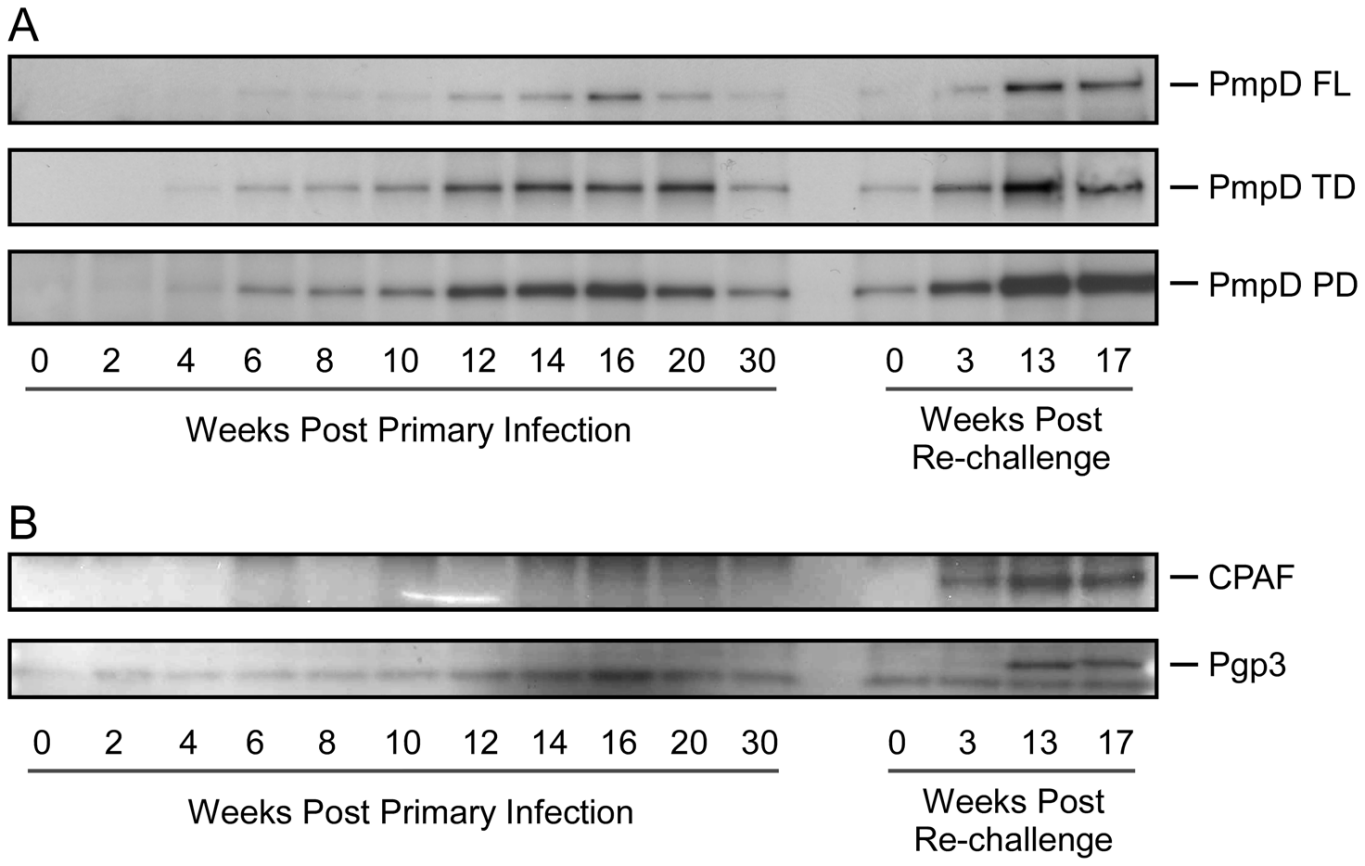
Identification of trachoma conserved antigens by western blotting using chlamydial antigen specific antibodies. IP was performed using monkey RML134 serum and chlamydial proteins prepared from non-denaturing detergent lysed non-radiolabeled A2497 infected McCoy cells. The IP proteins we identified by Western blot using a panel of rabbit monospecific antibodies generated against PmpD and mouse monoclonal antibodies specific to CPAF and Pgp3. (A) The higher MW polypeptides were identified as full length PmpD (155 kDa) and its proteolytically processed 82 kDa translocator and 73 kDa passenger domains [10] (B) Two lower MW polypeptides reacted with antibodies specific to CPAF and Pgp3.

To identify both high and low molecular mass antigenically conserved proteins shown in Figure 2 we employed a panel of monoclonal or monospecific polyclonal antibodies of known specificity to probe IP antigens recognized by NHP sera. Immunoprecipitation for this study was performed using sera from monkey RML 134 obtained during primary infection and following re-challenge using unlabeled chlamydial proteins prepared from non-denaturing detergent lysed infected McCoy cells. Using this procedure we identified some of the high and low molecular weight antigenically conserved proteins identified in Figure 2. Western blots of the IP proteins using a panel of PmpD anti-peptide antibodies identified full length PmpD and its two proteolytically processed fragments, the 82 kDa translocator domain and a 73 kDa passenger domain (Figure 3A) [10]. We also identified the 60 kDa polypeptide as Hsp60 (data not shown). We surmised that the low molecular weight proteins might be two previously described immunogenic chlamydial antigens, chlamydial protease-like activity factor (CPAF) and the plasmid encoded gene protein 3 (Pgp3). Both proteins have been shown to be secreted, antigenically conserved, and immunogenic by other investigators [11]–[15]. We identified the 29 kDa polypeptide as the C-terminal fragment of CPAF and the 25 kDa polypeptide as Pgp3 (Figure 3B). The other three polypeptides remain to be identified.

### Heat sensitivity of the antigens recognized by NHP antibodies

**Figure 4:**
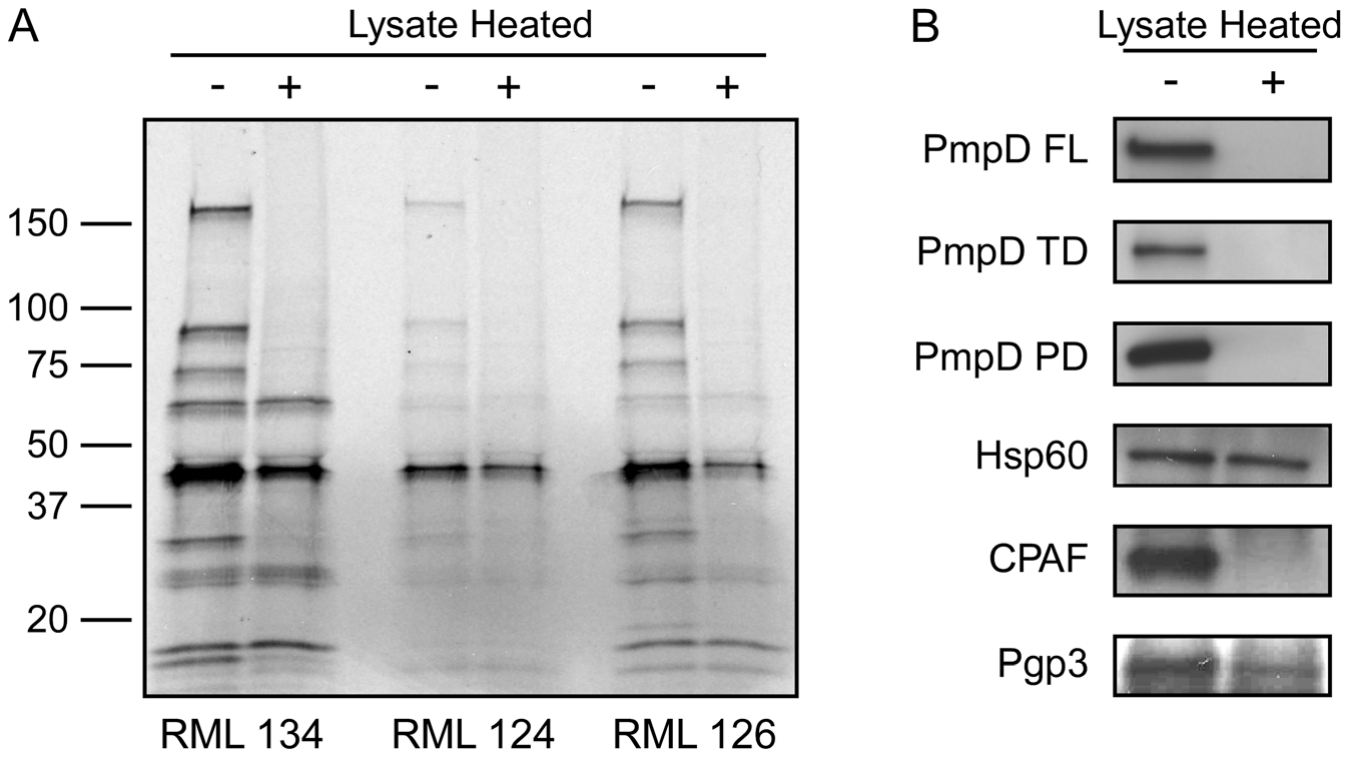
A majority of the antibodies generated against chlamydial specific and conserved antigens recognize conformational determinants. (A) RIP was performed with sera from all three infected NHP obtained from a single time point 13 weeks after re-challenge using A2497 infected cell lysates that were treated at 70°C for 30 min or kept at ambient temperature. Heat treatment of chlamydial antigens partially destroyed antibody recognition of MOMP in all three monkeys. Heating also destroyed or partially destroyed the antigenicity of the majority of the other IP antigens indicating that antibodies generated against the antigens in the context of primary and re-challenge infections were specific to conformational determinants. (B) IPs were performed on heated and unheated A2497 infected cell lysates using sera from monkey RML 134 and gels probed by Western blotting with chlamydial specific antibodies to identify heat sensitive and resistant conserved antigens. Heat treatment completely inhibited antibody reactivity against full length PmpD and its two proteolytically processed polypeptides and CPAF. Pgp3 and HSP60 antigenicity was partially affected or not affected by heating, respectively.

Next we studied whether antibody recognition of chlamydial proteins was sensitive to heat denaturation as an indirect parameter to differentiate between native conformational and contiguous epitopes. Sera from all three infected monkeys were incubated with unheated or heated (70°C for 30 minutes) RIP antigen (Figure 4). Antibody recognition of MOMP was significantly decreased which is consistent with our previous observation that NHP anti-MOMP antibodies are at least partially directed against conformational epitopes of the heat-labile MOMP trimer [5]. All three of the highest MW polypeptides were destroyed by heating. The 60 kDa polypeptide antigenicity was not noticeably affected by heat treatment. Two of the 4 low MW polypeptides were heat sensitive. To presumptively identify the heat sensitive proteins, IP were similarly performed on heated and unheated non-labeled A2497 lysates followed by Western blotting using antibodies against PmpD, Hsp60, CPAF and Pgp3 (Figure 4B). Antibody recognition of PmpD, its proteolytic fragments and CPAF was destroyed after heat treatment. To a lesser extent Pgp3 recognition was also destroyed, consistent with previous findings describing the heat stable nature of Pgp3 trimers and the observation that anti-Pgp3 antibodies reactive with the trimer react poorly with the denatured monomer [16]. In contrast, antibody recognition of Hsp60 was resistant to heat treatment.

## Discussion

In this study we investigated whether a long-duration but self-limiting ocular *C. trachomatis* infection in NHP primates was related to antigenic drift or whether gradual maturation of the host antibody mediated immune response was required to eradicate the infection. We detected no signs of antigenic drift in *ompA* or other chlamydial genes. This finding is consistent with epidemiological studies showing MOMP does not change rapidly even in trachoma endemic areas [17], [18]. However, we found that a slow maturation of the humoral immune response specific to multiple chlamydial antigens strongly correlates with reduction in early infection burdens, complete eradication of infection, and partial immunity to re-challenge.

In a study similar to ours, Caldwell et al. examined the temporal serum antibody response in cynomolgus macaques infected ocularly with *C. trachomatis* serovar B by western blotting [19]. In general, they found a simple temporal relationship in antigen recognition with a uniform and predominant response against the MOMP at approximately 21 days post-infection. A more variable and later appearance of antibody specific to chlamydial HSP60 and lipopolysaccharide was also detected; however antibody recognition was limited to these three antigens. Those findings agree with ours in part, but there are noteworthy differences between the studies both in experimental design and methodology. The Caldwell et al. study infected macaques ocularly with *C. trachomatis* serovar B, a laboratory reference strain of unknown virulence. The strain produced a self-limiting acute conjunctivitis that resolved spontaneously at approximately 8 weeks post-infection without delayed clearance or episodes of reactivation; no culture positivity was seen after week 6. The animals were not re-challenged following clearance of the primary infection so their protective immune status was unknown. Finally, western blotting was used to define anti-chlamydial antibody specificity over the infection period, a method that detects antibodies primarily against denatured protein antigens. In contrast, we employed a recent serovar A trachoma clinical isolate (A2497), a strain shown to have enhanced virulence characteristics for macaques compared to a laboratory adapted *C. trachomatis* serovar A strain (HAR-13) [7]. Ocular infection with A2497 resulted in a long-duration primary infection that did not spontaneously resolve until approximately 9–15 weeks post-challenge. Notably, A2497 long-duration infections produced repeated episodes of infection reactivity, modulating from culture negative to culture positive periods over the course of a primary infection that was accompanied by severe to moderate clinical disease. Collectively these results argue that A2497 infection of NHPs more closely mimics trachoma in humans [7]. We also re-challenged A2497 infected macaques after spontaneous clearance to evaluate protective immunity. Finally, and importantly, we employed RIP assay rather than Western blotting to study the humoral immune response to A2497 infection. The RIP assay utilizes intrinsically radiolabeled chlamydiae prepared from infected cells under non-denaturing conditions. This is a valid methodological difference as Western blotting measures only denatured antigen found in purified chlamydial EBs, whereas RIP assay is capable of detecting all chlamydial antigens associated with infected cells; these include antigens associated with both the EB and RB developmental forms of the organism and chlamydial secreted antigens. Thus, the larger spectrum and diversity of antigenic targets extracted under non-denaturing condition provides a unique opportunity to comprehensively study infection induced humoral immunity to conformationally dependent epitopes of these antigens. We argue that this is relevant and important to trachoma vaccine design. This hypothesis is supported by the findings that native MOMP is a superior immunogen for eliciting protective immunity in both the mouse and NHP [7], [20]. In another more recent study, Lu et al. investigated the antibody response in trachoma patients in a genome-wide scale [21]. Lu et al. used GST fused antigens expressed in a heterologous system and they describe recognition of multiple potentially important chlamydial proteins. However, their experiments failed to recognize both PmpD and MOMP, the two most immunogenic proteins in chlamydiae, likely because recognition of these antigens in humans is dependent on native confirmation.

Our findings imply that an efficacious trachoma vaccine might require multiple chlamydial antigens expressed in their native confirmation. A logical cocktail of recombinant antigens based on our results would consist of MOMP, PmpD, CPAF, and Pgp3, although other low molecular weight antigens associated with infection eradication that were not identified in this study may also be needed. Mechanistically, the kinetics of antibodies specific for these proteins infers that antibody specific to MOMP, a surface exposed and antibody neutralizing target, likely function in reducing early chlamydial burdens, whereas antibodies to CPAF, Pgp3 and PmpD, another surface exposed neutralizing target, are required to eradicate infection. This hypothesis is consistent with previous reports using these antigens as single subunit vaccines in both NHP and murine models. Kari et al. immunized NHP with native-MOMP extracted from *C. trachomatis* EBs that resulted in a significant 70 fold reduction in infectious ocular burdens early post infection [5]. Interestingly, native-MOMP immunization had no effect on the duration of the infection suggesting that complete clearance of ocular chlamydial infection requires additional non-MOMP related antigens. In numerous studies employing recombinant CPAF and Pgp3 as immunogens in mice, protection was repeatedly limited to later time periods post-challenge with no to minimal effect on reducing early chlamydial burdens or shedding [13], [22]–[27]. A caveat of our study is that we cannot conclude antibodies are the primary protective mechanism as they might simply be surrogate markers of protective T cell immune responses. It is unclear why the *C. trachomatis* specific humoral immune response takes so long to fully develop. It is possible that recognition of certain potentially protective epitopes requires long-term antigen stimulation. This would be similar to the natural protective immunity that older individuals develop in hyper endemic areas after years of long-term exposure through repeated reinfection episodes. Nevertheless, we believe a vaccine containing a cocktail of these antigens might generate protective immunity capable of functioning at early and late time points during chlamydial infection. A vaccine with these protective properties would be beneficial in preventing or reducing chlamydial transmission and re-infection, both of which are driving forces in the pathology of blinding trachoma. The difficulty however with this approach is if the native conformation of these proteins is critical to protective immunity, as our findings imply, producing these immunogens as recombinant proteins in heterologous expression systems will be a major yet worthy challenge. An alternative vaccine approach that would negate this requirement is the use of a live-attenuated trachoma vaccine, such as the recently described live-attenuated plasmidless vaccine [28]. The advantage of a live-attenuated vaccine is that it naturally presents multiple antigens to the mucosal immune system in their native conformation. This not only allows appropriate recognition by B cells to generate antibody against native antigens but confers the ability of the host to process and present these antigens in association with class I and II HLA molecules that may be equally important for T cell immunity. If successful, a multivalent subunit vaccine or an efficacious live attenuated vaccine could ultimately prevent hundreds of thousands cases of conjunctival scarring and blindness.
